# Lack of sex-related analysis and reporting in Cochrane Reviews: a cross-sectional study

**DOI:** 10.1186/s13643-021-01867-3

**Published:** 2022-12-26

**Authors:** Alba Antequera, M. Ana Cuadrado-Conde, Emilia Roy-Vallejo, María Montoya-Martínez, Montserrat León-García, Olaya Madrid-Pascual, Sara Calderón-Larrañaga, on behalf of Research for gender equity collaboration

**Affiliations:** 1 Biomedical Research Institute Sant Pau, Hospital de la Santa Creu i Sant Pau, Barcelona, Spain; 2 Accident and Emergency Department, King’s College Hospital, London, UK; 3 Internal Medicine Department, Hospital Universitario de La Princesa, Madrid, Spain; 4 Servicio Murciano de Salud, Coordinación Estratégica para la Cronicidad Avanzada y Atención Sociosanitaria, Murcia, Spain; 5 Arztpraxis Kalkbreite, Zürich, Switzerland; 6 Centre for Primary Care and Public Health, Queen Mary University of London, London, UK

**Keywords:** Gender bias, Systematic reviews, Equity, External validity, Reporting

## Abstract

**Background:**

Sex-specific analysis and reporting may allow a better understanding of intervention effects and can support the decision-making process. Well-conducted systematic reviews (SRs), like those carried out by the Cochrane Collaboration, provide clinical responses transparently and stress gaps of knowledge. This study aimed to describe the extent to which sex is analysed and reported in a cross-section of Cochrane SRs of interventions, and assess the association with the gender of main authorships.

**Methods:**

We searched SRs published during 2018 within the Cochrane Database of Systematic Reviews. An investigator appraised the sex-related analysis and reporting across sections of SRs and collected data on gender and country of affiliation of the review first and last authors, and a second checked for accuracy. We conducted descriptive statistics and bivariate logistic regression to explore the association between the gender of the authors and sex-related analysis and reporting.

**Results:**

Six hundred and ten Cochrane SRs were identified. After removing those that met no eligibility criteria, 516 reviews of interventions were included. Fifty-six reviews included sex-related reporting in the abstract, 90 considered sex in their design, 380 provided sex-disaggregated descriptive data, 142 reported main outcomes or performed subgroup analyses by sex, and 76 discussed the potential impact of sex or the lack of such on the interpretations of findings. Women represented 53.1 and 42.2% of first and last authorships, respectively. Women authors (in first and last position) had a higher possibility to report sex in at least one of the review sections (OR 2.05; CI 95% 1.12–3.75, *P*=0.020) than having none.

**Conclusions:**

Sex consideration amongst Cochrane SRs was frequently missing. Structured guidance to sex-related analysis and reporting is needed to enhance the external validity of findings. Likewise, including gender diversity within the research workforce and relevant authorship positions may foster equity in the evidence generated.

## Background

Since the beginning of medicine and across disciplines, white males have been considered the biological standard for all health conditions, and research on nonreproductive health of the female population has been traditionally ignored [1, 2]. Female participants have, by extension, been underrepresented and overlooked not only in medical schools but also in basic and clinical research [3–7]. For example, in the USA after institutional efforts of the National Institutes of Health (NIH) over the last several decades [8, 9], the percentage of females included in clinical trials funded by the NIH reached up to 50% [10]. Nevertheless, an increase of female representation in study samples has not led to an improvement in the analysis and data disaggregation by sex or gender [11, 12], i.e., ‘gender bias’ [13], and it may carry avoidable risks, especially for the underrepresented population. For example, sex/gender analyses may guide specific recommendations based on differential effects for sexes, as tailored drug doses or relation benefits-harms of certain procedures [14, 15].

Sex and gender are interrelated concepts but not synonyms. Sex refers to biological traits, whereas gender is based on socially constructed features [16]. Although genetic, cellular, biochemical, and physiological differences between males and females have been described for decades [17, 18], and several initiatives from grant agencies and journals and editors are underway to tackle the scarce attention to sex and gender in biomedical science [19–22], there remains a lack of sex and gender integration in evidence production, both in primary studies and systematic reviews (SRs) [23, 24].

The Cochrane Library is a collection of databases that contain high-quality, independent SRs to inform clinical practice and health policy. The Cochrane Equity Methods Group recently developed a tool to help review authors to improve sex and gender reporting in their SRs [25]. However, Cochrane SRs might not be exempt from gender bias [26]. Meanwhile, a recent study suggested that the presence of women in main authorship positions might improve the degree of sex and gender consideration in research [27]. Biological and social-based differences lead to differential health risks, disease incidence, and health service needs [28]. Thus, a systematic assessment of sex-related reporting and analysis may support different approaches of intervention for specific patient groups (especially, when the benefit-harm balance remains unclear for certain interventions) and highlight gaps of knowledge for further more inclusive medical research. We described the extent to which sex is analysed and reported in Cochrane SRs of interventions published in 2018, and assessed the relationship between sex-related analysis and reporting and gender of main authorships.

## Methods

### Eligibility criteria

We included Cochrane reviews on intervention studies. We excluded reviews that involved single-sex because they addressed sex-specific health conditions, and those that had been withdrawn from publication. We restricted the publication date to 2018 to remain the reference numbers at manageable levels for fast human screening and data extraction.

### Identification of Cochrane reviews and data extraction

We searched for Cochrane reviews using the advanced search option within the Cochrane Database of Systematic Reviews from January 1, 2018, to December 31, 2018. Two authors (from AA, ACC, ERV, MLG, SCL) independently screened titles and abstracts, and when appropriate, full-texts. We developed and pre-tested a data extraction form in Excel to collect data on gender and country affiliation of first and last review author, and sex- and gender-related analysis and reporting in SRs (i.e., review authors used any sex or gender-related terms to provide information across the SR). One author (from AA, ACC, ERV, MLG, SCL) extracted data on sex-related reporting in the following review sections: abstract, methods, results, and discussion. This manual inspection also involved the Control-F search command to look for keywords such as “male”, “female”, “women”, “men”, “woman”, “man”, “sex” and “gender”. A second author (from AA, ACC, ERV, MLG, OMP, SCL) checked data extraction for accuracy verification. For the results section, we made the difference between a descriptive assessment, as sex-disaggregated data reported either in the main text or table of characteristics of included studies, and analytic approaches, as sex-disaggregated results from pooling data from primary studies through the main meta-analysis or subgroups analyses. We rated as non-applicable in the results and discussion sections assessment, those reviews that reported no studies meeting the eligibility criteria or included insufficient studies to perform pre-specified analyses. We determined the gender of the first and last review authors by name when provided a reasonable reliance on the perceived gender of the investigators; otherwise, we used Gender-API software [29]. In the case of names that might be assigned for both genders or assignation remained doubtful, we grouped the given name, family name, and affiliation institution details and tracked it on the institution website and social media. In this paper, we accepted the sex and gender terminology used by the review author regardless of the appropriateness of the terms [30, 31]. Thus, hereafter, we used the “sex/gender” to mean sex and/or gender” terms for participants in reviews and the “gender” term for authors.

### Data analysis

We described the frequencies for sex/gender reporting in each of the aforementioned review sections, the gender of authorship in dominant positions, and author’s country of affiliation categorised according to the World Bank country income index [32]. We dealt with missing and non-applicable data by listwise deletion to calculate frequencies. We reported data as medians, percentages, and ranges. We performed different logistic models depending upon the review section to assess the odds of sex/gender-related reporting. We did not use the income index as a confounder to avoid ecological fallacy [33]. We established statistical significance at *P* value of 0.05. We performed statistical analyses using STATA statistical software (version 13; College Station, USA).

## Results

### Selection of Cochrane reviews

**Fig. 1:**
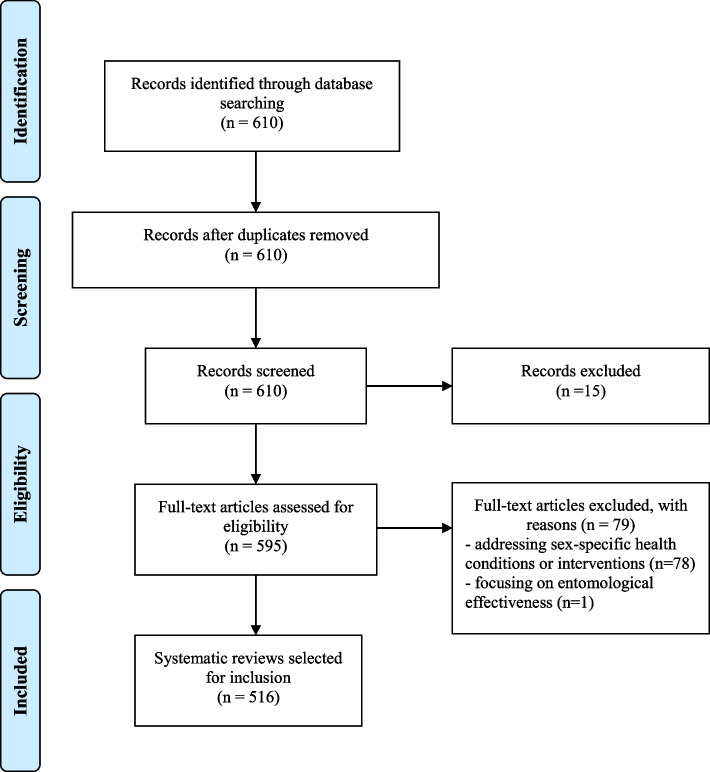
Flow diagram showing the selection of systematic reviews

We identified 610 Cochrane SRs with a publication date of 2018. We excluded 94 reviews from further analyses due to withdrawn publication (15 SRs), addressing sex-specific interventions (78 SRs), and testing the entomological effectiveness of long-lasting insecticidal nets (1 SR) [34]. Finally, we included 516 reviews of interventions in our analyses. Figure 1 represents the PRISMA flow diagram.

### Characteristics of included Cochrane reviews

Two-thirds of included Cochrane reviews were updated versions. Four publications were individual publication data systematic reviews [35–38]. Nine reviews were signed by collaborative research groups, and seven of them were conducted by the same group. The median number of authors was 5 (range 2–22). We identified 1,031 main authorship positions since one collaborative research group in one review was considered as a single signature. Women were first or last authors in 53.1% and 42.2% of the reviews, respectively. In 25.78% (133/516 SRs) of the cases, both authors were women, and in 69.57% (359/516 SRs), at least one of the main authors was women. Although authors were widely distributed geographically, no authors were affiliated to institutions in low-income countries. Most authors (874; 84.7%) came from high-income countries, over one-third of them (378; 43.2%) based on the UK.

### Sex/gender-related analysis and reporting in Cochrane reviews

Table 1 shows the overall sex/gender-related analysis reporting in each of the sections assessed. Overall, 10.9% (56/516 SRs) of reviews included sex-related reporting in abstract sections, and 17.4% (90/516 SRs) considered sex in their methods. We judged 30 reviews to be non-applicable for the results section assessment. Of the remaining, 78.2% (380/486 SRs) of reviews presented sex-disaggregated descriptive data, while 31.9% (142/445 SRs) amongst those that could pool data reported using an analytic approach by sex (i.e., main outcomes or performed subgroup analyses by sex). As for the discussion section, we rated 16 reviews as non-applicable, whereas 15.2% (76/500 SRs) of reviews reflected on the potential impact of sex (or the lack of such) on the clinical and research implications. In addition, after pooling review sections, we found that 40.3% (208/516 SRs) of reviews reported on sex in at least two sections, and 2.7% (14/516 SRs) did it in all five sections. In the individual participant data (IPD) SR subset, we detailed that only one [35] out of the four IPD reviews reported both sex-disaggregated descriptive and analytic results and encouraged caution in the use of drugs assessed in the female group, while the others only reported descriptive results by sex.

**Table 1:** Sex/gender-related analysis and reporting in Cochrane reviews

Review section	Sex/gender reporting, % (*N*)	Item was not applicable to SR, % (*N*)
Abstract	10.9 (56)	0
Methods	17.4 (90)	0.2 (1)
Descriptive results	78.2 (380)^a^	5.8 (30)
Analytic results	31.9 (142)^b^	13.8 (71)
Discussion	15.2 (76)^c^	3.3 (17)
At least two sections	40.3 (208)	0
At least three sections	16.3 (84)	0
All sections	2.7 (14)	0

Abbreviations: *SR* systematic review

^a^Denominator: Number of SRs that were applicable (*N*=486)

^b^Denominator: Number of SRs that were applicable (*N*=445)

^c^Denominator: Number of SRs that were applicable (*N*=500)

We analysed distribution of frequencies between sex/gender reporting and gender authorships as shown in Table 2. First woman authorship significantly improved sex/gender reporting in the abstract section (*P*=0.019). SRs with woman in the last authorship positions showed a significant sex/reporting in descriptive results (*P*=0.027). Amongst reviews in which both authors were women, 39.3% and 35.5% of the SRs reported on sex/gender in the abstract (*P*=0.015) and discussion (*P*=0.046), respectively.

**Table 2:** Sex/gender reporting by review section according to gender of authors

Sex/gender reporting in each review section	First author (female)		Last author (female)		Both authors female^a^		At least one author female	
	% (***N***)	***P*** value	% (***N***)	***P*** value	% (***N***)	***P*** value	% (***N***)	***P*** value
Abstract (*N*=56)	68% (38)	0.019	44.6% (25)	0.701	39.3% (22)	0.015	73.2% (41)	0.531
Methods (*N*=90)	55.6% (50)	0.623	50.0% (45)	0.105	28.8% (26)	0.465	76.7% (69)	0.114
Descriptive results (*N*=380)	56.1% (213)	0.147	45.0% (171)	0.027	28.1% (107)	0.127	72.9% (277)	0.012
Analytic results (*N*=142)	51.4% (73)	0.465	45.8% (65)	0.405	30.3% (43)	0.316	66.9% (95)	0.386
Discussion (*N*=76)	60.5% (46)	0.195	50.0% (38)	0.161	35.5% (27)	0.046	75.0% (57)	0.315
At least two sections (*N*=208)	56.7% (118)	0.175	45.2% (94)	0.266	29.3% (61)	0.130	72.6% (151)	0.221
At least three sections (*N*=84)	60.7% (51)	0.126	51.2%(43)	0.070	36.9% (31)	0.011	75.0% (63)	0.238
All sections (*N*=14)	57.1% (8)	0.759	57.1% (8)	0.253	35.7% (5)	0.389	78.6% (11)	0.459

^a^Both authors are women compared to at least one being a man

We carried out logistic regression to assess the possibility of reporting sex/gender depending on gender authorship (number of women authors in dominant positions) as shown in Table 3. SRs in which both authors were women had a higher odd of sex/gender reporting than SRs with none women authors in the following comparisons: descriptive results section (OR 1.98, 95% CI 1.11–3.55, *P*=0.021), and, after pooling any of the assessed sections, in at least one section (OR 2.05, 95% CI 1.12–3.75, *P*=0.020), and three or more sections (OR 1.97, 95% CI 1.07–3.62, *P*=0.030). For descriptive results having one woman as an author also increased the possibility of sex/gender reporting (OR 1.65, 95% CI 1.09–2.70, *P*=0.046). Other comparisons found no significant associations; however, they showed a positive and progressive association for the number of women authors and sex reporting.

**Table 3:** Logistic regression sex/gender reporting by review section according to gender of authors

Review section	OR	CI 95%	***P*** value
**Abstract**			
One woman author	0.87	0.43–1.77	0.698
Two women authors	1.88	0.67–1.87	0.079
**Methods**			
One woman author	1.51	0.86– 2.66	0.154
Two women authors	1.56	0.83–2.93	0.164
**Descriptive results**			
One woman author	1.65	1.09–2.70 *	0.046
Two women authors	1.98	1.11–3.55 *	0.021
**Analytical results**			
One woman author	0.71	0.44–1.14	0.160
Two women authors	1.03	0.62–1.73	0.904
**Discussion**			
One woman author	1.08	0.58–2.01	0.793
Two women authors	1.78	1.12–3.75	0.079
**1 or more sections**			
One woman author	1.27	0.78– 2.05	0.333
Two women authors	2.05	1.12–3.75 *	0.020
**2 or more sections**			
One woman author	1.16	0.76–1.77	0.486
Two women authors	1.49	0.93–2.38	0.099
**3 or more sections**			
One woman author	1.07	0.59–1.93	0.827
Two women authors	1.97	1.07–3.62 *	0.030
**All sections**			
One woman author	1.4	0.34–5.68	0.638
Two women authors	2.00	0.47–8.55	0.347

One woman: one woman in a main position authorship. Two women: two women in a main position authorship

*Abbreviations*: *CI* confidence interval, *OR* odds ratio

## Discussion

Our findings showed that overall sex/gender consideration in Cochrane reviews was inadequate, given that only 2.7% of the SRs reported it in all five sections. When the first and last authors were women, there was a higher possibility of sex/gender-related reporting. We also found a lack of representation of reviews produced in low-income countries.

Our results are consistent with previous findings on insufficient sex/gender reporting in SRs [24, 31]. Interestingly, abstracts showed the lowest sex/gender reporting of all sections (10.9%). This is both consistent with previous work [26] and significant, especially considering that abstracts are supposed to showcase the most relevant findings of a SR. Reporting in the methods section was also inadequate (17.4%). Concerning the results section, we found that sex/gender reporting was highest in the descriptive section (78.2%), and consistently lower for the analytic approaches (31.9%). Taken together, the repeatedly lower ratio of reporting on analytical items that we and others observed [24, 26] suggests a concerning lack of attention to the potential role of sex/gender in the study outcomes. In view with this, Morgan et al. enhanced some considerations about the conception, development and data management which would foster gender-related analysis in health system research [39]. The known insufficient data about sex/gender in primary studies [11, 12, 40] hinders its reporting in SRs. However, when that is the case, the discussion section gives authors the possibility to address such limitations. Still, we showed that only 15.2% of SRs addressed participants’ sex/gender in their discussions. Clearly, when sex/gender reporting is not considered a priority, the lack of the necessary data will likely be overlooked.

This generalised lack of sex/gender consideration is a matter of concern, which has led to the publication of relevant reporting guidelines and support tools by internationally known research institutions, such as the Cochrane Collaboration, NIH or Canadian Institutes for Health Research [8, 16, 41]. However, despite policy and institutional support, our results show that the problem remains.

We described a link between first and last women’s authorship and the possibility of a sex/gender-related analysis and reporting. Nielsen et al. provided strong evidence associating sex/gender-related analysis with the presence of women in the author group, especially in leading first and last author positions, using a sample of more than 1.5 million medical research papers [27]. A recent bibliometric analysis examining more than 11.5 million studies also showed an increased probability of reporting sex/gender in articles where the first and last authors were women [23]. Our findings, therefore, contribute to the existing debate on the relevance of women’s participation in science, as a means of counteracting and even reversing institutional cultures that promote sex-blind research [1].

In relation to the country of origin, no review was conducted by authors affiliated to low-income countries. In contrast, Sugimoto and colleagues found poorer rates of sex/gender-related reporting in North America, compared to under-resourced research settings, such as Africa [23]. Larivière and colleagues found higher prevalence of women’s authorship in countries with lower scientific output, with South America and Eastern Europe being the regions with greatest gender parity [41]. In our case, the scarcity of authors affiliated to low- and middle-income countries has hampered the analysis of any relevant variations.

Our study has several caveats and limitations. First, we did not analyse whether sex/gender reporting was more likely in disease-specific research areas as described previously [40]. Second, external validity of SRs goes beyond sex/gender and is influenced by other axes of inequity (and intersections between them) that have not been considered in this study [9, 42, 43]. Third, we acknowledge that our binary approach to sex and gender and the assignment of gender based on authors’ names can be both problematic and inaccurate [44, 45]. Further, we used sex and gender terms interchangeably and combined (‘sex/gender’) when referring to the degree of reporting in Cochrane SRs. However, we acknowledge that sex and gender are different concepts [16]. Our decision was pragmatic and shaped by the frequently inaccurate use of these terms in primary studies and Cochrane SRs. Finally, although we collected countries of affiliation, we used no this variable as a confounder in order to avoid the ecological fallacy [33]. Moreover, we collected no additional variables that may have been confounders, so we have not adjusted our models. In terms of strengths, the number of SRs included in our study, as well as the peer reviewed rating system gave consistency to our research findings. To our knowledge, this is the first study investigating the association between sex/gender reporting and gender of authorship amongst Cochrane SRs. The fact that Cochrane SRs are used worldwide to support evidence-based clinical practice and policy, make our findings of particular relevance, with the potential to inform high-quality research within Cochrane collaboration, and support rigorous decision making worldwide.

Further Cochrane systematic reviews should enhance adherence to international reporting guidelines on sex and gender equity [30, 46]. The Cochrane Collaboration has developed structured guidance and tools tailoring to sex- and gender-based analysis [24, 47], while they may require amendments to be consistent with the current standards of conducting systematic reviews, including health equity in the GRADE approach [48], when appropriate. Researchers, editors, and funding organisations need to demand better sex- and gender-related analysis and reporting to achieve the purpose of conducting methodologically sound systematic reviews.

## Conclusions

Sex- and gender-blind research contributes to biased decisions in policymaking and health service provision. In order to avoid this, our research suggests that sex and gender must be taken into account throughout the lifecycle of research. This involves diversifying both the scientific workforce and research populations, promoting appropriate methodological approaches for the analysis of data and reinforcing regulation that ensures that the twin goals of diversity and excellence in science are met.

## Data Availability

The data that support the findings of this study was derived from the following resources available in the public domain: https://www.cochranelibrary.com/cdsr/reviews. However, all the information is available from the corresponding author upon reasonable request. Alba Antequera is a doctoral candidate in Public Health and Methodology of Biomedical Research, at the Department of Pediatrics, Obstetrics, Gynaecology and Preventive Medicine at Universitat Autònoma de Barcelona (Spain) and this work is part of her PhD.

## Abbreviations

CI: Confidence interval
NIH: National Institutes of Health
OR: Odds ratio
SR: Systematic reviews
